# The Emerging Roles of LINC00665 in Human Cancers

**DOI:** 10.3389/fcell.2022.839177

**Published:** 2022-03-09

**Authors:** Jing Zhu, Yirao Zhang, Xuyu Chen, Yibo Bian, Juan Li, Keming Wang

**Affiliations:** Department of Oncology, Second Affiliated Hospital, Nanjing Medical University, Nanjing, China

**Keywords:** lncRNA, LINC00665, cancer, biomarker, therapeutic target

## Abstract

Long non-coding RNAs (lncRNAs) are non-coding RNAs that have more than 200 nucleotides and can participate in the regulation of gene expression in various ways. An increasing number of studies have shown that the dysregulated expression of lncRNAs is related to the occurrence and progression of human cancers. LINC00665 is a novel lncRNA, which is abnormally expressed in various human cancers, such as lung cancer, breast cancer, prostate cancer, and glioma. LINC00665 functions in many biological processes of tumor cells, such as cell proliferation, migration, invasion, angiogenesis, and metabolism, and is related to the clinicopathological characteristics of cancer patients. LINC00665 can play biological functions as a ceRNA, directly binding and interacting with proteins, and as an upstream molecule regulating multiple signaling pathways. In this review, we comprehensively summarize the expression level, function, and molecular mechanisms of LINC00665 in different human cancers and emphasize that LINC00665 is a promising new diagnostic, prognostic biomarker, and therapeutic target.

## Introduction

Cancer is the leading cause of death and an essential obstacle to increasing life expectancy in every country of the world (Sung et al., 2021). Worldwide, an estimated 19.3 million new cancer cases and almost 10.0 million cancer deaths occurred in 2020 (Sung et al., 2021). Although there are multiple treatments, including surgery, radiotherapy, chemotherapy, and targeted drugs, their effectiveness remains limited (Chi et al., 2019). Therefore, there is an urgent need to find novel and effective cancer biomarkers and therapeutic targets that are more sensitive for early diagnosis, treatment, and prognosis of human cancers than traditional biomarkers and methods.

LncRNAs are RNAs longer than 200 nucleotides in length (Goodall and Wickramasinghe, 2021). Like mRNAs, lncRNAs are transcribed by polymerase II, mainly 5′capped, polyadenylated, and spliced, but in general, they contain fewer exons than mRNAs (Schmitz et al., 2016). They comprise a heterogeneous class of intergenic transcripts, enhancer RNAs, and sense or antisense transcripts that overlap other genes (Kopp and Mendell, 2018). More and more evidence shows that lncRNAs play complex and precise regulatory roles in the occurrence and development of human cancers by acting as oncogenes or tumor suppressors (Tan et al., 2021). They act as pivotal signal transduction mediators in cancer signal pathways by interacting with proteins, RNAs, and lipids (Lin and Yang, 2018). Existing studies have revealed that lncRNAs are involved in a variety of biological processes, including tumor cell proliferation (Wang et al., 2020a), apoptosis (Zhao et al., 2020a), epithelial to mesenchymal transition (EMT) (McCabe and Rasmussen, 2021), migration (Dhamija and Diederichs, 2016), invasion (Dhamija and Diederichs, 2016), drug resistance (Wei et al., 2020), angiogenesis (Zhao et al., 2020b), and metabolism (Lin, 2020). Although many studies have revealed the expression level and function of lncRNAs in human cancers, the detailed regulatory mechanisms of many lncRNAs in the occurrence and progression of human cancers are still unknown.

**FIGURE 1:**
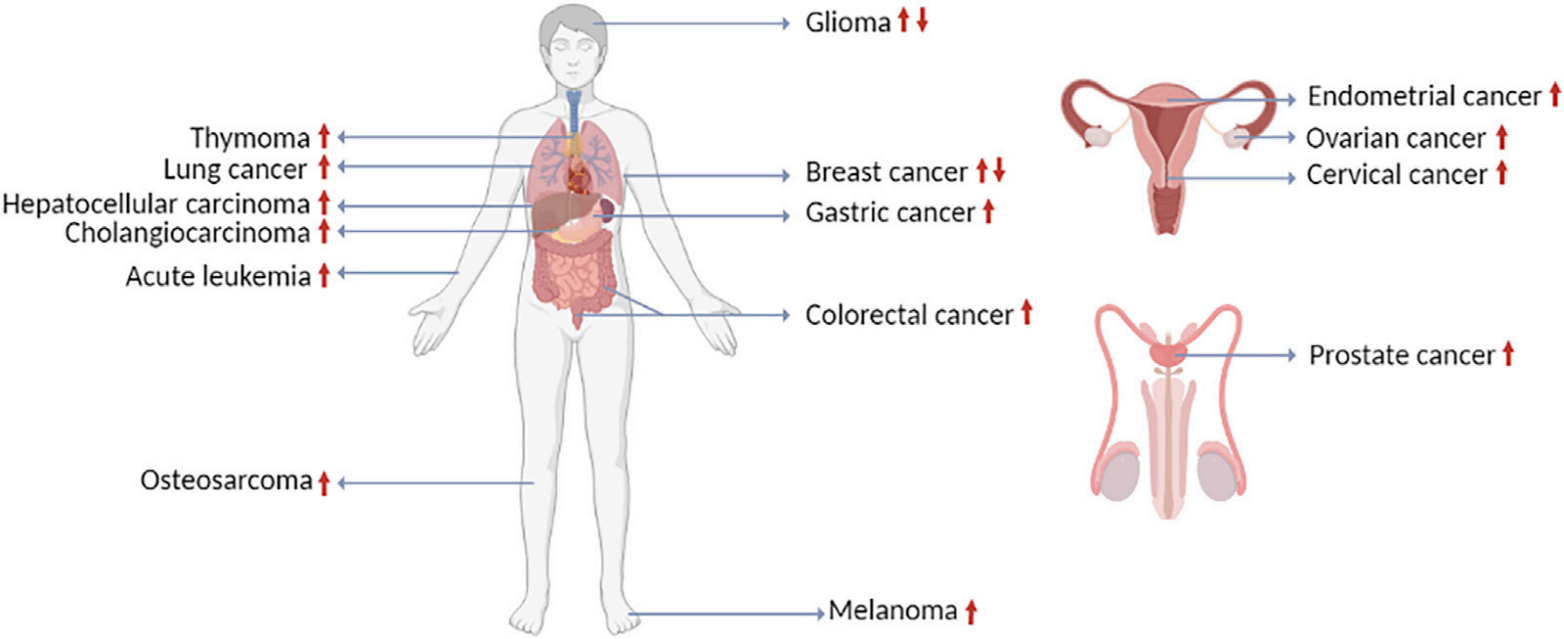
The expression of LINC00665 in human cancers (created with BioRender.com).

LINC00665 (also called CIP2A-BP) is a novel lncRNA located on chromosome 19q13.12 and abnormally expressed in many cancers (Figure 1). It has two different transcripts: LINC00665-1 (1749 bp, transcript variant 1) and LINC00665-2 (988 bp, transcript variant 2). LINC0066 is abnormally expressed in multiple human cancers and acts as an oncogene or tumor suppressor gene, including lung cancer, breast cancer, prostate cancer, glioma, hepatocellular carcinoma, colorectal cancer, ovarian cancer, gastric cancer, cholangiocarcinoma, acute leukemia, endometrial cancer, melanoma, cervical cancer, and thymoma (Table 1). This review summarizes the literature on the expression level, roles, and related molecular mechanisms of Linc00665 in cancers, thus pointing out the clinical potential of Linc00665 as a diagnostic, prognostic biomarker, and therapeutic target.

**TABLE 1:** The functional characterization of LINC00665 in human cancers.

Cancer type	Property	Expression	Function	Related genes/proteins/pathways	References
Lung cancer	oncogene	upregulated	proliferation, migration, invasion, apoptosis, cell cycle, EMT, angiogenesis, drug resistance	miR-138-5p/E2F3; miR-181c-5p/ZIC2; miR-let-7b/CCNA2, miR-98/AKR1B10/ERK; miR-195-5p/MYCBP/c-MYC, EZH2/CDKN1C; EZH2/PI3K/AKT; YB-1	Cong et al. (2019), Liu et al. (2019), Wang et al. (2020b), Cong et al. (2020), Wang et al. (2021a), Yang et al. (2021a), Huang et al. (2021), Wei et al. (2021)
Breast cancer	oncogene	upregulated	proliferation, migration, invasion, apoptosis, EMT	miR-379-5p/LIN28B; miR-551b-5p, miR-3619-5p/CTNNB1/β-catenin	Ji et al. (2020), Lv et al. (2020), Zhou et al. (2020), Qi et al. (2021)
	suppressor	downregulated	migration, invasion	TGF-β/Smad/4E-BP1/elF4E/CIP2A-BP/CIP2A/PP2A/PI3K/AKT/NF-κB	Guo et al. (2020)
Prostate cancer	oncogene	upregulated	proliferation, migration, invasion	miR-1224-5p/SND1; EZH2, LSD1/KLF2	Chen et al. (2020), Eke et al. (2021), Xue et al. (2021)
Glioma	oncogene	upregulated	proliferation, migration, invasion, cell cycle, glycolysis	miR-34a-5p/AGTR1	Dai et al. (2021a), Wang et al. (2021b)
	suppressor	downregulated	proliferation, migration, invasion, apoptosis	TAF15/LINC00665/STAU1/MTF1(YY2)/GTSE1	Ruan et al. (2020)
Hepatocellular carcinoma	oncogene	upregulated	proliferation, migration, invasion, apoptosis, autophagy, glycolysis	miR-186-5p/MAP4K3; miR-214-3p/MAPK1, NF-κB/LINC00665/PKR/NF-κB	Wen et al. (2018), Shan and Li (2019), Ding et al. (2020), Wan et al. (2021)
Colorectal cancer	oncogene	upregulated	proliferation, migration, invasion, apoptosis, cell cycle	miR-9-5p/ATF1; miR-126-5p/PAK2, FZD3, miR-214-3p, U2AF2/CTNNB1/Wnt/β-catenin	(Zhao et al., 2020c; Han et al., 2021; Wu et al., 2021)
Ovarian cancer	oncogene	upregulated	proliferation, migration, invasion, autophagy	miR-34a-5p/E2F3; miR-146a-5p/CXCR4	Gao et al. (2020), Meng et al. (2020), Wu et al. (2020), Xu et al. (2021)
Gastric cancer	oncogene	upregulated	proliferation, migration, invasion, apoptosis, cell cycle, EMT	miR-379-5p/GRP78; miR-149-3p/RNF2, Wnt/β-catenin; TGF-β	Qi et al. (2019), Yang et al. (2020), Yue et al. (2021), Zhang and Wu (2021)
Cholangiocarcinoma	oncogene	upregulated	proliferation, migration, invasion, apoptosis, EMT, drug resistance	miR-424-5p/BCL9L/Wnt/β-catenin	Lu et al. (2021)
Osteosarcoma	oncogene	upregulated	proliferation, migration, invasion	miR-3619; miR-708, miR-142-5p/RAP1B	Zhang et al. (2020), Wang et al. (2021c)
Acute myeloid leukemia	oncogene	upregulated	proliferation, migration, Apoptosis, adhesion	miR-4458/DOCK1/Rac1	Yang et al. (2021b)
Acute lymphoblastic leukemia	oncogene	upregulated	proliferation, migration, invasion	miR-101/PI3K/Akt	Abuduer and A, (2021)
Endometrial cancer	oncogene	upregulated	proliferation, migration, invasion, apoptosis, cell cycle	HMGA1	Cai et al. (2021)
Melanoma	oncogene	upregulated	proliferation, migration	miR-224-5p/VMA21	Wang et al. (2020c)
Cervical cancer	oncogene	upregulated	proliferation, migration, invasion, EMT	CTNNB1/Wnt/β-catenin	Xia et al. (2021b)
Thymoma	oncogene	upregulated	—	miR-140/MYO10; miR-3199/WASF3	Chen et al. (2021b)

## Function of LINC00665 in Cancers and Underlying Molecular Mechanisms

### Lung Cancer

Lung cancer is the second most common cancer and the leading cause of cancer morbidity and mortality in men. Among women, it ranks third in incidence and second in mortality (Sung et al., 2021). Tobacco smoking is the primary risk factor for lung cancer (Yoshida et al., 2020). Most patients with lung cancer are only diagnosed when the disease is already at an advanced stage (Wadowska et al., 2020). Therefore, early diagnosis is crucial to improve the overall prognosis of lung cancer patients. Many studies have demonstrated that lncRNAs play a fundamental role in multiple cell processes of lung cancer and can be used as biomarkers of lung cancer (Chen et al., 2021a).

Wang et al. (2020b) used a lncRNA microarray to analyze paired lung cancer tissues and adjacent normal lung tissues and found that LINC00665 was markedly upregulated in tumor tissues. Similarly, Cong et al. (2019) used qRT-PCR analysis to measure LINC00665 expression in 80 paired lung cancer tissues and corresponding normal tissues and found that LINC00665 was upregulated in lung cancer tissues. Furthermore, they also demonstrated that the transcription factor SP1 could bind to the promoter of LINC00665 and partly upregulate the transcription of LINC00665 in lung cancer cells (Cong et al., 2019). Yang et al. (2021a) revealed that the high expression of LINC00665 was associated with advanced TNM stage, lymph node metastasis, and tumor size. The Kaplan-Meier survival analysis showed that patients with high LINC00665 expression had shorter overall survival and progression-free survival than patients with low LINC00665 expression (Cong et al., 2019; Yang et al., 2021a; Wei et al., 2021). Researchers used FISH and qRT-PCR to investigate the subcellular location of LINC00665, and the results revealed that LINC00665 was predominately located in the cytoplasm (Wang et al., 2021a). Researchers performed flow cytometry, CCK-8 assays, and transwell assays to evaluate the effects of LINC00665 on lung cancer cell apoptosis, proliferation, migration, and invasion (Cong et al., 2019; Wang et al., 2020b; Wang et al., 2021a; Yang et al., 2021a; Wei et al., 2021). The high expression of linc00665 promoted the proliferation, migration, invasion of lung cancer cells *in vitro* and modulated cell cycle arrest and apoptosis (Cong et al., 2019; Wang et al., 2020b; Wang et al., 2021a; Yang et al., 2021a; Wei et al., 2021).

Mechanically, Wang et al. proved the direct interaction between miR-138-5p and LINC00665 through bioinformatics analysis and the luciferase reporter assay (Wang et al., 2020b). Compared with normal lung tissues, miR-138-5p expression in lung cancer tissues was relatively low, and the expression of miR-138-5p and LINC00665 were significantly negatively correlated (Wang et al., 2020b). They next used bioinformatics analysis and found that miR-138-5p may bind to E2F3 3′UTR (Wang et al., 2020b). Then they performed the luciferase reporter assay *in vitro* to verify this result (Wang et al., 2020b). When they artificially downregulated the expression of LINC00665, E2F3 expression protein was suppressed. In conclusion, they revealed that LINC00665 upregulated E2F3 expression by acting as a competing endogenous RNA (ceRNA) for miR-138-5p, thereby promoting lung cancer progression (Wang et al., 2020b). Similarly, Wei et al. (2021) concluded that Linc00665 could bind with miR-181c-5p to upregulate ZIC2, thereby promoting the growth and invasion of lung cancer cells. Huang et al. (2021) determined that LINC00665/miR-let-7b/CCNA2 was an essential ceRNA network related to the prognosis of lung cancer. Cong et al. (2019) demonstrated that LINC00665 reinforced the proliferation and invasion of lung cancer cells *in vitro* and *in vivo* by functioning as a ceRNA for miR-98 and subsequently activating the AKR1B10-ERK signaling pathway. Another study proved that LINC00665 could act as a ceRNA by sponging miR-195-5p to upregulate MYCBP, activating the canonical c-MYC pathway in lung cancer (Wang et al., 2021a). Studies have also reported that the high expression of LINC00665 was related to drug resistance in lung cancer (Liu et al., 2019; Yang et al., 2021a). Yang et al. (2021a) revealed that knockdown of LINC00665 enhanced the sensitivity of lung cancer cells to cisplatin *in vitro* and *in vivo*. Further studies have reported that LINC00665 could recruit EZH2 to the promoter region of CDKN1C to inhibit its transcription (Yang et al., 2021a). What’s more, they proved that CDKN1C overexpression inhibited the proliferation and migration of lung cancer cells and enhanced the sensitivity of lung cancer cells to cisplatin (Yang et al., 2021a). Liu et al. (2019) found thatLINC00665 expression was significantly upregulated in lung cancer tissues and gefitinib-resistant cells. Moreover, they certified that LINC00665 could drive gefitinib resistance by increasing EZH2 and activating the PI3K/AKT pathway (Liu et al., 2019). In addition, another study showed that LINC00665 interacted with YB-1 and promoted angiogenesis in lung cancer (Cong et al., 2020).

### Breast Cancer

Breast cancer has surpassed lung cancer as the leading cause of global cancer incidence in 2020. It is the fifth leading cause of cancer mortality worldwide, with approximately 2.3 million new cases and 685,000 deaths (Sung et al., 2021). Therefore, it is necessary to explore further the pathogenesis and therapeutic targets of breast cancer.

Lu et al. analyzed the expression level of LINC00665 in breast cancer tissues and matched normal tissues in the database (Lv et al., 2020). They found that the expression of the LINC00665 was significantly upregulated in breast cancer tissues (Lv et al., 2020). Subsequently, they used qRT-PCR to identify LINC00665 expression in 106 pairs of breast cancer tissues and adjacent normal tissues and also proved that LINC00665 was highly expressed in breast cancer tissues (Lv et al., 2020). Furthermore, LINC00665 expression was significantly associated with the tumor size and TNM stage, but not with the age of the breast cancer patients (Lv et al., 2020). In addition, Qi et al. conducted the Kaplan-Meier survival analysis to explore the correlation between LINC00665 expression and the prognosis of breast cancer patients (Qi et al., 2021). They found that high expression of LINC00665 could predict poor overall survival of breast cancer patients (Qi et al., 2021). Studies have reported that LINC00665 played a vital role in breast cancer progression, and LINC00665 Knockdown inhibited breast cancer cells proliferation, migration, and invasion but promoted apoptosis (Ji et al., 2020; Lv et al., 2020; Zhou et al., 2020; Qi et al., 2021). Moreover, Ji et al. (2020) examined the expression of mesenchymal and epithelial markers by qRT-PCR, Western blot, and immunofluorescence. They found that in breast cancer cells and mouse tumor tissues with high expression of LINC00665, E-cadherin was downregulated, and Vimentin and N-cadherin were significantly upregulated, indicating that LINC00665 induced EMT-like phenotype in breast cancer cells (Ji et al., 2020). Similarly, Zhou et al. (2020) proved that LINC00665 could promote the progression of EMT in breast cancer cells. In order to further investigate the potential mechanism of LINC00665 carcinogenesis, Ji et al. (2020) determined that LINC00665 was mainly expressed in the cytoplasm by using fluorescence *in situ* hybridization (FISH) and subcellular separation followed by qRT-PCR. Since cytoplasmic lncRNAs could function as miRNA sponges by competitively binding common miRNAs, they used the luciferase reporter assay and the RIP assay to prove the direct binding relationship between LINC00665 and miR-379-5p (Ji et al., 2020). Furthermore, they predicted that LIN28B was a target of miR-379-5p by using starBase (Ji et al., 2020). They conducted the luciferase reporter assay to prove their binding relationship (Ji et al., 2020). Additionally, they observed that overexpression of miR-379-5p resulted in the downregulation of LIN28B in breast cancer cells (Ji et al., 2020). Taken together, they concluded that overexpression of LINC00665 promoted LIN28B expression via sponging miR-379-5p (Ji et al., 2020). Similarly, Lv et al. (2020) demonstrated that LINC00665 acted as a miR-3619-5p sponge and inhibited tumorigenesis by regulating β-catenin expression. Qi et al. (2021) showed that the LINC00665/miR-551b-5p axis was involved in breast cancer progression.

LncRNAs usually do not encode proteins, but recent studies have shown that some of these lncRNAs could encode biologically active micropeptides involved in various cellular activities (Guo et al., 2020). Guo et al. (2020) revealed that LINC00665 could encode a micropeptide called CIP2A-BP. The expression of CIP2A-BP was downregulated in triple-negative breast cancer, and low expression of CIP2A-BP was associated with poor survival in triple-negative breast cancer patients (Guo et al., 2020). In exploring the downregulation mechanism, they found that the TGF-β signaling pathway could affect the translation process of CIP2A-BP. Specifically, the Smad signaling pathway activated by TGF-β induced the expression of translation inhibitory protein 4E-BP1, which inhibited eukaryote translation initiation factor elF4E, resulting in the downregulation of CIP2A-BP (Guo et al., 2020). *In vitro* knockdown and overexpression studies confirmed that CIP2A-BP, not the LINC00665 transcript, acted as a tumor suppressor gene in the development and progression of triple-negative breast cancer (Guo et al., 2020). Mechanistically, CIP2A-BP competed with PP2A to bind to CIP2A, thereby releasing PP2A activity that inhibited the PI3K/AKT/NFκB pathway (Guo et al., 2020). In summary, they found that in triple-negative breast cancer, TGF-β downregulated the translation of CIP2A-BP to induce tumor invasion and metastasis through CIP2A/PP2A and PI3K/AKT/NF-κB signaling pathway (Guo et al., 2020).

### Prostate Cancer

Prostate cancer is the second most frequent cancer and the fifth leading cause of cancer death among men in 2020 (Sung et al., 2021). The development of prostate cancer is usually very slow, and it may take a long time to develop into metastatic status (Lin et al., 2020). But most of the newly diagnosed prostate cancer patients are usually at the advanced stage with distant metastasis (Lin et al., 2020). Some studies have revealed that lncRNAs enable early cancers detection, and lncRNAs may also become therapeutic targets for prostate cancer patients (Flippot et al., 2019).

Xue et al. (2021) found that LINC00665 was significantly upregulated in prostate cancer tissues and cell lines, and high expression of LINC00665 was correlated with the higher T stage and lymph node metastasis of prostate cancer patients. In addition, they performed the Kaplan-Meier survival analysis and found that the high LINC00665 expression level was associated with the poor survival of prostate cancer patients (Xue et al., 2021). Furthermore, experiments have shown that LINC00665 knockdown inhibited the proliferation, migration, and invasion of prostate cancer cells (Chen et al., 2020; Xue et al., 2021). In the process of exploring the mechanism, Chen et al. (2020) found a negative correlation between miR-1224-5p and LINC00665 in prostate cancer. Moreover, they performed the RIP assay and the luciferase reporter assay to confirm the direct interaction between LINC00665 and miR-1224-5p (Chen et al., 2020). They further verified that SND1 was a downstream target of miR-1224-5p (Chen et al., 2020). Through qRT-PCR analysis, they found that LINC00665 silencing suppressed the expression of SND1, but the suppression of miR-1224-5p reversed it (Chen et al., 2020). Finally, they concluded that LINC00665 upregulated the expression of SND1 by inhibiting miR-1224-5p and ultimately promoted the progression of prostate cancer (Chen et al., 2020). Xue et al. (2021) conducted subcellular fractionation assays to confirm that LINC00665 was mainly located in the nucleus of prostate cancer cells, which indicated that LINC00665 might exert regulatory effects at transcriptional levels. Then, they confirmed that LINC00665 could directly bind with EZH2 and LSD1 in prostate cancer cells (Xue et al., 2021). They conducted ChIP analysis in prostate cancer cells and demonstrated that LINC00665 could recruit EZH2 and LSD1 to the KLF2 promoter region, leading to trimethylation of H3K27 or demethylation of H3K4 at this region (Xue et al., 2021). These results revealed that LINC00665 could inhibit the expression of KLF2 by interacting with EZH2 and LSD1 to promote prostate cancer progression (Xue et al., 2021). In particular, one study showed that LINC0065 was steadily upregulated in prostate cancer cells 2 months after radiotherapy (Eke et al., 2021). This study proved that LINC00665 regulated RBBP8, XPC, and BRCA1 gene expression (Eke et al., 2021). BRCA1 and RBBP8 encode the DNA repair protein CtIP, part of the homologous recombination pathway, while XPC is involved in nucleotide excision repair (Eke et al., 2021). In addition, silencing of LINC00665 reduced and delayed radiation-induced RAD51 formation. These results indicated that LINC00665 modulate DNA repair pathways (Eke et al., 2021).

### Glioma

Glioma is the most common and malignant primary tumor of the central nervous system, and it is a complex and heterogeneous tumor (Cheng et al., 2020a). Although many treatments, such as surgery, radiotherapy, and chemotherapy, glioma is still a complex disease to treat (Cheng et al., 2020b). Some studies have shown that the abnormal expression of lncRNAs may play an important role in glioma (Xia et al., 2021a).

Dai et al. (2021a) revealed the high expression of LINC00665 in glioma tumor tissues through lncRNA microarray and qRT-PCR analysis. Subsequently, the Kaplan-Meier survival analysis determined that the high expression of LINC00665 was related to the unsatisfactory overall survival of glioma patients (Dai et al., 2021a). The CCK-8 assay, the cell migration assay, and the cell invasion assay revealed that reduced expression of LINC00665 could decrease the proliferation, migration, and invasion of glioma cells (Dai et al., 2021a). In addition, qRT-PCR results showed that LINC00665 was distributed in the cytoplasm fraction of glioma cells (Dai et al., 2021a). Analysis using the starBase bioinformatics prediction database demonstrated that sequences in miR-34a-5p were significantly similar to the LINC00665 3′untranslated region (UTR) (Dai et al., 2021a). The luciferase reporter assay and the RIP assay results indicated that miR-34a-5p could bind to LINC00665 *in vitro* (Dai et al., 2021a). Similarly, the researchers proved that AGTR1 was a target gene of miR-34a-5p (Dai et al., 2021a). Finally, their research results indicated that LINC00665 could modulate AGTR1 expression by sponging miR-34a-5p, thus modulating glioma growth (Dai et al., 2021a). In particular, a study confirmed that glycolysis-related LINC00665 could impact poor prognosis, cell proliferation, invasion, cell cycle, and metastasis through the biological process of glycolysis, and the risk model based on glycolysis-related LINC00665 could significantly predict prognosis and might be used as a therapeutic target (Wang et al., 2021b).

Contrary to its tumor-promoting effect, LINC0665 has been reported to exert a tumor suppressor effect in glioma (Ruan et al., 2020). Ruan et al. (2020) revealed that LINC00665 expression was downregulated in glioma tissues compared with that in normal brain tissues and was negatively correlated with the pathological grade of glioma. Moreover, they confirmed that upregulation of LINC00665 expression inhibited the proliferation, migration, and invasion of glioma cells and promoted their apoptosis (Ruan et al., 2020). Similarly, they found that TAF15 expression was downregulated in glioma tissues and cells (Ruan et al., 2020). The interaction between TAF15 and LINC00665 was confirmed by the RIP assay and the RNA pull-down assay (Ruan et al., 2020). Furthermore, they proved that overexpression of TAF15 stabilized LINC00665, thus increasing its expression level (Ruan et al., 2020). Further research indicated that LINC00665 could destabilize MTF1 and YY2 mRNA by interacting with STAU1, and knockdown of STAU1 could rescue the MTF1 and YY2 mRNA degradation caused by LINC00665 overexpression (Ruan et al., 2020). They next evaluated the potential modulatory effect of MTF1 and YY2 on GTSE1 (Ruan et al., 2020). Western blot and qRT-PCR showed that overexpression of MTF1 and YY2 increased the mRNA and protein expression level of GTSE1, while knockdown of MTF1 and YY2 reduced GTSE1 level (Ruan et al., 2020). In summary, their study confirmed that overexpression of TAF15 stabilized LINC00665, thereby increasing its expression level to reduce the STAU1-mediated mRNA degradation of both MTF1 and YY2, further inhibiting the transcription of GTSE1 and ultimately destroying the malignant progression of glioma (Ruan et al., 2020).

### Hepatocellular Carcinoma

Hepatocellular carcinoma is the sixth most commonly diagnosed cancer and the third leading cause of cancer death worldwide in 2020 (Sung et al., 2021). Although the incidence of the disease has decreased, the disease-specific mortality rate remains high (Yang and Heimbach, 2020). Emerging evidence has recently suggested the crucial role of lncRNAs in the tumorigenesis and progression of hepatocellular carcinoma (Huang et al., 2020a).

Wen et al. (2018) extracted LINC00665 data from TCGA that could be used for expression analysis. Data analysis results showed that in 370 confirmed cases of hepatocellular carcinoma, the expression of LINC00665 in tumor tissues was significantly increased compared with matched normal liver tissues. Shan and Li (2019) performed qRT-PCR analysis to detect the expression of LINC00665 in 76 pairs of hepatocellular carcinoma tissues and matched normal adjacent tissues, which also proved the high expression of LINC00665 in hepatocellular carcinoma tissues. In addition, Ding et al. analyzed the relationship between LINC00665 RNA level and clinicopathological characteristics in 122 pairs of hepatocellular carcinoma and adjacent non-cancerous tissues (Ding et al., 2020). They found that LINC00665 expression was positively correlated with the TNM stage and Barcelona Clinic Liver Cancer (BCLC) stage (Ding et al., 2020). Furthermore, Wan et al. (2021) used the Kaplan-Meier survival analysis to confirm that the overall survival of hepatocellular carcinoma patients with high LINC00665 expression was shorter than those with low LINC00665 expression. Experiments *in vitro* proved that downregulation of LINC00665 inhibited the proliferation, migration, and invasion of hepatocellular carcinoma cells and induced apoptosis and autophagy (Shan and Li, 2019; Wan et al., 2021).

Experiments including the luciferase reporter assay, the RIP assay, and the RNA pull-down assay have clarified the direct interaction between LINC00665 and miR-186-5p (Shan and Li, 2019). In addition, MAP4K3 depletion inhibited cell viability and induced apoptosis and autophagy, indicating that MAP4K3 may act as an oncogene in the progression of hepatocellular carcinoma (Shan and Li, 2019). Researchers also observed that the downregulation of miR-186-5p attenuated the tumor suppressor effect of MAP4K3 knockdown in hepatocellular carcinoma cells (Shan and Li, 2019). In conclusion, researchers revealed that LINC00665 could upregulate MAP4K3 expression by sponging miR-186-5p, thereby promoting the progression of hepatocellular carcinoma (Shan and Li, 2019). Moreover, Wan et al. (2021) found that LINC00665 upregulation caused a significant increase in ATP levels, lactate fraction, and glucose consumption of hepatocellular carcinoma cells. In contrast, LINC00665 knockdown reduced glucose consumption, lactate fraction, and ATP levels (Wan et al., 2021). These results indicated that LINC00665 promoted aerobic glycolysis in hepatocellular carcinoma (Wan et al., 2021). In terms of mechanism, they pointed out that LINC00665 was overexpressed in HCC, which accelerated cell growth and migration and triggered aerobic glycolysis through sponging miR-214-3p to increase MAPK1 expression (Wan et al., 2021). Another study clarified that NF-κB signaling induced LINC00665 and LINC00665 interacted with PKR and exerted its carcinogenic activity by promoting the activation and stability of PKR, thereby providing feedback on NF-κB signaling (Ding et al., 2020). The discovery of the NF-κB/LINC00665/PKR/NF-κB loop provided a way to understand the connection between inflammation and cancer (Ding et al., 2020).

### Colorectal Cancer

The incidence of colorectal cancer is the third, and mortality is the second (Sung et al., 2021). Age, genetic and environmental factors play a role in colorectal cancer (Thanikachalam and Khan, 2019). It is worth noting that the incidence of colorectal cancer in young people is rising (Keum and Giovannucci, 2019). Emerging evidence has indicated that lncRNAs play a crucial role in the occurrence and progression of colorectal cancer (Chen and Shen, 2020).

Wu et al. (2021) measured the expression of LINC00665 by qRT-PCR in 67 colorectal cancer tissues and their matched adjacent healthy tissues. They found significant upregulation of LINC00665 in colorectal cancer tissues compared to healthy control tissues. In addition, Zhao et al. (2020c) further analyzed the relationship between the expression of LINC00665 and the clinicopathological indicators of colorectal cancer. They found that the high expression of LINC00665 in tumor tissues was significantly related to regional lymph node metastasis and poor colorectal cancer tissue differentiation in patients. Experiments *in vitro* revealed that LINC00665 knockdown could inhibit the proliferation, migration, and invasion of colorectal cancer cells and induce apoptosis in colorectal cancer cells (Zhao et al., 2020c; Han et al., 2021; Wu et al., 2021). Mechanistically, researchers used StarBase to predict the targeted miRNA of LINC00665 and found that miR-9-5p was one of the candidate targets of LINC00665 (Zhao et al., 2020c). Subsequently, they verified the binding site between LINC00665 and miR-9-5p through bioinformatics analysis, the luciferase reporter assay, and the RIP assay (Zhao et al., 2020c). To further investigate the downstream molecular mechanism of miR-9-5p, they used TargetScan to predict the target gene of miR-9-5p and found that ATF1 was a candidate target gene of miR-9-5p (Zhao et al., 2020c). Subsequent experimental results also confirmed this prediction (Zhao et al., 2020c). They conclude that LINC00665 promoted the progression of colorectal cancer by regulating the miR-9-5p/ATF1 axis (Zhao et al., 2020c). Another study found that LINC00665 could upregulate the expression of PAK2 and FZD3 through sponging miR-126-5p (Wu et al., 2021). Han et al. (2021) found that LINC00665 could activate the Wnt/β-catenin signaling pathway by upregulating the expression of CTNNB1, ultimately stimulating the tumorigenicity of colorectal cancer. When they explored the mechanism of LINC00665 regulating CTNNB1, they found that LINC00665 could upregulate the expression level of CTNNB1 through sponging miR-214-3p or by binding to U2AF2 protein to enhance the stability of CTNNB1 mRNA (Han et al., 2021).

### Ovarian Cancer

Ovarian cancer is the third most common gynecologic malignancy worldwide but accounts for the highest mortality rate among these cancers (Kuroki and Guntupalli, 2020). Most women with ovarian cancer are already at advanced stages when diagnosed (Huang et al., 2020b). At present, the treatment options available are very limited. Therefore, there is an urgent need for new effective treatment strategies.

Xu et al. (2021) found that LINC00665 was upregulated in ovarian cancer tissues and cell lines and correlated to overall survival and progression-free survival of ovarian cancer. In addition, the level of LINC00665 was related to tumor size, FIGO stage, and lymph node metastasis (Xu et al., 2021). Through the CCK-8 assay and the colony formation assay, they found that knockdown of LINC00665 reduced proliferative rate in ovarian cancer cells (Xu et al., 2021). Besides, the migration and invasion ability of ovarian cancer cells decreased after LINC00665 was downregulated (Xu et al., 2021). Bioinformatic prediction depicted a binding sequence in miR-34a-5p 3′UTR pairing to LINC00665 (Xu et al., 2021). They found that miR-34a-5p was significantly downregulated in ovarian cancer tissues, and when LINC00665 was knocked down in breast cancer cells, miR-34a-5p was upregulated (Xu et al., 2021). These results revealed that LINC00665 regulated ovarian cancer progression by targeting miR-34a-5p as a ceRNA (Xu et al., 2021). By comprehensive analysis of online databases, they predicted that E2F3 was a downstream gene of miRNA-34a-5p, which was further verified by the luciferase reporter assay (Xu et al., 2021). In conclusion, they revealed that LINC00665 promoted ovarian cancer progression by regulating the miRNA-34a-5p/E2F3 axis (Xu et al., 2021). Similarly, with a series of integrated bioinformatics databases, Gao et al. (2020) systematically explored and identified lncRNAs related to ovarian cancer prognosis. Based on the ceRNA hypothesis, they successfully constructed a new ceRNA regulatory network in ovarian cancer: LINC00665/miR-146a-5/CXCR4 (Gao et al., 2020). Meng et al. (2020) conducted a comprehensive analysis of autophagy-related lncRNAs, obtained clinical data of ovarian cancer from TCGA, and identified 17 autophagy-related lncRNAs, including LINC00665. Moreover, Wu et al. (2020) found that the high expression of LINC00665 was correlated with the level of lymphocyte infiltration in breast cancer.

### Gastric Cancer

Gastric cancer ranks fifth for incidence and fourth for mortality globally (Sung et al., 2021). It is a very aggressive malignant tumor, with heterogeneity, and still a global health problem (Machlowska et al., 2020). The regulation of lncRNAs expression and the roles of lncRNAs in the progression and metastasis of gastric cancer have been widely discussed (Yuan et al., 2020).

Through a meta-analysis based on GEO and TCGA databases, Zhang et al. found that in 909 gastric cancer samples and 237 non-tumor samples, the expression of LINC00665 in tumor samples was significantly higher than that in non-tumor samples (Zhang and Wu, 2021). The Kaplan-Meier survival analysis showed that the high expression of LINC00665 was associated with poor overall survival and disease-free survival rate in gastric cancer patients (Qi et al., 2019; Zhang and Wu, 2021). Zhang and Wu (2021) also confirmed the correlation between LINC00665 expression and tumor depth, lymph node metastasis, and TNM stage in gastric cancer. Moreover, studies have determined that the upregulation of LINC00665 could promote the proliferation, invasion, and migration of gastric cancer cells and inhibit cell apoptosis (Qi et al., 2019; Yang et al., 2020; Zhang and Wu, 2021). To determine the potential mechanism of LINC00665 in the progression of gastric cancer, Qi et al. (2019) used online bioinformatics analysis to predict the potential target of LINC00665. They found that miR-149-3p was a target of LINC00665 (Qi et al., 2019). QRT-PCR showed that LINC00665 inhibition significantly increased the expression of miR-149-3p in gastric cancer cells (Qi et al., 2019). In addition, the luciferase reporter assay and the pull-down assay suggested that LINC00665 could regulate miR-149-3p expression *via* a ceRNA manner (Qi et al., 2019). They further confirmed that RNF2 was a downstream target of miR-149-3p through the luciferase reporter assay (Qi et al., 2019). In summary, their research revealed that the LINC00665/miR-149-3p/RNF2 axis was involved in gastric cancer progression (Qi et al., 2019). Zhang and Wu (2021) detected the EMT-related proteins by Western blot analysis. They found that downregulation of LINC00665 could significantly reduce the mesenchymal-related proteins N-cadherin and vimentin in gastric cancer cells, whereas inducing the epithelial-related protein E-cadherin (Zhang and Wu, 2021). In addition, they also found that the expression levels of TGF-β, Smad-2, and α-SMA were reduced, so they concluded that LINC00665 could promote the progress of EMT in gastric cancer cells by activating TGF-β and its downstream signaling pathway (Zhang and Wu, 2021). Yang et al. (2020) demonstrated that inhibited LINC00665 could decrease the β-catenin and CyclinD1 protein expression in gastric cancer cells and induce GSK-3β protein level. Therefore, they reported that LINC00665 could be used as a critical oncogene in gastric cancer progression by activating the Wnt signaling pathway (Yang et al., 2020). In particular, Yue et al. (2021) revealed the regulatory effects of LINC00665 on cisplatin resistance in gastric cancer. They demonstrated that LINC00665 silencing increased the sensitivity of cisplatin by inhibiting endoplasmic reticulum stress (Yue et al., 2021). Specifically, LINC00665 could sponge miR-379-5p to upregulate GRP78, thereby promoting the resistance of gastric cancer cells to cisplatin (Yue et al., 2021).

### Cholangiocarcinoma

Cholangiocarcinoma is the second most common primary liver cancer after hepatocellular carcinoma (Rodrigues et al., 2021). It is usually asymptomatic in the early stages, diagnosed in the late stage (Rodrigues et al., 2021). Cholangiocarcinoma includes a group of highly heterogeneous malignant tumors of the biliary tract, which can occur at any point of the biliary tree (Banales et al., 2020). Due to multiple mutations or other factors, patients often develop drug resistance (Sato et al., 2020). The mechanism of drug resistance in cholangiocarcinoma needs to be further explored.

Lu et al. (2021) established two cholangiocarcinoma cell lines resistant to gemcitabine and identified dysregulated lncRNAs through lncRNA microarray. They found that LINC00665 was significantly upregulated in gemcitabine-resistant cholangiocarcinoma cell lines (Lu et al., 2021). Next, they further checked the expression of LINC00665 in 100 pairs of cholangiocarcinoma samples and matched adjacent normal tissues (Lu et al., 2021). The results showed that LINC00665 was upregulated in cholangiocarcinoma patients, consistent with the previous results (Lu et al., 2021). In addition, they concluded that the high expression of LINC00665 was positively correlated with higher TNM stage, lymph node metastasis, and distant metastasis in cholangiocarcinoma patients (Lu et al., 2021). In the Kaplan-Meier survival analysis, the overall survival time and recurrence-free survival time of cholangiocarcinoma patients with high LINC00665 expression were significantly shortened (Lu et al., 2021). Furthermore, they reported that LINC00665 knockdown increased the cytotoxic activity of gemcitabine on cell apoptosis and growth, thereby weakening gemcitabine tolerance of resistant cholangiocarcinoma cells (Lu et al., 2021). They conducted the sphere formation assay, the cell invasion assay, and the cell migration assay and found that silencing LINC00665 inhibited gemcitabine-induced EMT and stemness properties in resistant cholangiocarcinoma cells (Lu et al., 2021). Mechanically, they demonstrated that LINC00665 could upregulate BCL9L expression by acting as a molecular sponge for miR-424-5p, which subsequently increased the nuclear translocation of β-catenin and Wnt signal activation, thus increasing the stemness of gemcitabine-induced EMT and resistant cholangiocarcinoma cells (Lu et al., 2021).

### Osteosarcoma

Osteosarcoma is a highly aggressive cancer and the most common form of bone cancer in children and young adults (Sayles et al., 2019). Although surgical resection and neoadjuvant chemotherapy reduce the mortality of osteosarcoma patients, the 5-year survival rate remains low (Wang et al., 2019). Therefore, it is essential to find new diagnostic biomarkers and therapeutic targets.

Recent studies have found that the expression of Linc00665 was significantly upregulated in osteosarcoma samples than in normal samples, which was related to tumor size and tumor stage (Wang et al., 2021c). In addition, the overall survival time of patients with high expression of LINC00665 was shorter than that of patients with low expression of LINC00665 (Zhang et al., 2020). Moreover, the CCK8 assay, the wound-healing assay, and the transwell assay exhibited that the overexpression of LINC00665 promoted the proliferation, migration, and invasion of osteosarcoma cells (Zhang et al., 2020; Wang et al., 2021c). Zhang et al. (2020) utilized the luciferase reporter assay and the RIP assay to prove that LINC00665 was directly related to miR-3619. Furthermore, miR-3619 expression was negatively correlated with the expression level of LINC00665, and silencing LINC00665 could increase miR-3619 expression (Zhang et al., 2020). Subsequently, they found that the upregulation of miR-3619 inhibited the viability, invasion, and migration of osteosarcoma cells, while these effects were offset by the overexpression of LINC00665 (Zhang et al., 2020). These results indicated that linc00665 promoted the progression of osteosarcoma through sponging miR-3619 (Zhang et al., 2020). Similarly, another study also proved that LINC00665 could function as a miRNA sponge for miR-708 and miR-142-5p (Wang et al., 2021c). Besides, LINC00665 upregulated the RAP1B expression via miR-708 and miR-142-5p (Wang et al., 2021c). RAP1B was essential for LINC00665 that exercised its biological functions of promoting proliferation, migration, and invasion (Wang et al., 2021c).

### Acute Leukemia

Leukemia is a heterogeneous hematological malignancy caused by uncontrolled neoplastic proliferation of undifferentiated or partially differentiated hematopoietic cells (Nabavizadeh et al., 2016). Acute leukemia is divided into acute myeloid leukemia (AML) and acute lymphoblastic leukemia (ALL) and is the most common hematological tumor in young people (Di Martino et al., 2021). It is crucial to uncover the pathogenesis of acute leukemia and explore novel strategies for acute leukemia treatment.

Yang et al. (2021b) revealed that LINC00665 was significantly upregulated in AML tissues and cell lines. Correlation analysis showed that the expression of LINC00665 was negatively correlated with miR-4458 in AML bone marrow tissue, and the results of qRT-PCR also displayed that miR-4458 was downregulated in AML tissue (Yang et al., 2021b). The luciferase reporter assay further proved that LINC00665 and miR-4458 could directly interact in AML cells (Yang et al., 2021b). Moreover, they demonstrated that LINC00665 promoted the proliferation, adhesion, and migration of AML cells but restricted the apoptosis of AML cells by sponging miR-4458 (Yang et al., 2021b). Their findings also revealed that the expression of DOCK1 in AML tissues was abnormally upregulated and that it showed a negative correlation with the expression of miR-4458 (Yang et al., 2021b). The results of the luciferase reporter assay and the RNA pull-down assay confirmed that miR-4458 could directly target DOCK1 (Yang et al., 2021b). In addition, they demonstrated that DOCK1 could promote AML progression by activating Rac1 (Yang et al., 2021b). Similarly, another study indicated that LINC00665 was highly expressed in ALL (Abuduer and A, 2021). Overexpression of LINC00665 promoted the viability, migration, and invasion of ALL cells, whereas silencing of LINC00665 did oppositely (Abuduer and A, 2021). Furthermore, overexpressed LINC00665 could promote ALL progression by targeting miR-101 to activate the PI3K/Akt pathway (Abuduer and A, 2021).

### Other Cancers

Recent experiments have proved that LINC00665 also played a significant role in other cancers, including endometrial cancer (Cai et al., 2021), melanoma (Wang et al., 2020c), cervical cancer (Xia et al., 2021b), and thymoma (Chen et al., 2021b). In endometrial cancer, Cai et al. used qRT-PCR and found that LINC00665 was elevated in endometrial carcinoma compared to normal endometrial tissues (Cai et al., 2021). Mechanically, LINC00665 promoted the occurrence and progression of endometrial cancer by interacting with HMGA1 (Cai et al., 2021). In melanoma, Wang et al. (2020c) reported that Linc00665 was significantly upregulated in melanoma tissues. Functional assays indicated that downregulation of LINC00665 inhibited the proliferation and invasion of melanoma cells (Wang et al., 2020c). Mechanically, they pointed that LINC00665 induced its oncogenic role via melanoma by upregulating VMA21 through sponging miR-224-5p (Wang et al., 2020c). In cervical cancer, Xia et al. indicated that LINC00665 regulated the proliferation, migration, invasion, and EMT of cervical cancer cells through the WNT-CTNNB1/β-catenin signaling pathway (Xia et al., 2021b). Chen et al. used bioinformatics analysis to reveal that LINN00665 was also highly expressed in thymoma (Chen et al., 2021b).

## Potential Clinical Application of LINC00665

Cancer survival rates are usually low because of late diagnosis and limited access to timely and standard treatment (Wu and Qu, 2015). Therefore, early and accurate detection of cancers is very significant for clinical diagnosis, effective toxicity monitoring, and ultimately successful treatment of cancers (Wu and Qu, 2015). Liquid biopsy refers to the analysis of tumor-derived biomarkers isolated from the biological fluids of cancer patients (Yamada et al., 2019). This approach is more accessible and less invasive (Goyal et al., 2021). LncRNAs dysregulation in primary tumor tissues is reflected in various body fluids, including whole blood, plasma, urine, saliva, and gastric juice (Bolha et al., 2017). In addition, although body fluids contain a large number of ribonucleases, researchers have found that lncRNAs could be detected and could resist the degradation activity of ribonucleases (Shi et al., 2016). These characteristics of lncRNAs provide the basis for lncRNAs to become effective and convenient diagnostic biomarkers. The LINC00665 described in this review is abnormally expressed in various human cancers and may become a new marker for cancer diagnosis.

Existing studies also show that the abnormal expression of LINC00665 is closely related to the prognosis of many human cancers. The dysregulated expression of LINC00665 is closely associated with clinicopathological results (including larger tumor size, lymph node metastasis, distant metastasis, and advanced clinical stage), overall survival rate, and recurrence-free survival rate. In particular, Dai et al. (2021b) revealed that breast cancer patients with high expression of Linc00665 were less likely to achieve pathological complete response after neoadjuvant chemotherapy, especially for HR-positive/HER2-negative patients. They pointed out that LINC00665 could become a new biomarker for the prognosis of breast cancer chemotherapy. In short, the previous research makes us believe that LINC00665 has the potential to become a prognostic marker of human cancers.

Increasing evidence suggests that lncRNAs play crucial roles in the occurrence and development of cancers and may become a therapeutic target (Toden et al., 2021). Wang et al. (2021a) designed short hairpin RNAs (shRNAs) that could specifically target LINC00665, and then they subcutaneously injected lung cancer cells transfected with sh-NC or sh-LINC00665 into nude mice. They found that sh-LINC00665 significantly suppressed tumor growth and lung metastasis in mice (Wang et al., 2021a). Similarly, Cai et al. (2021) conducted experiments *in vitro* and found that knocking down LINC00665 could inhibit the proliferation, migration, and invasion of endometrial cancer cells and induce apoptosis in endometrial cancer cells. Cong et al. (2020) also pointed out that compared with the control group, the supernatant from lung cancer cells with LINC00665 knockdown showed a significantly negative effect on the proliferation of vascular endothelial cells. Therefore, we fully believe that LINC00665 may become a therapeutic target for human cancers in the near future.

## Discussion

**FIGURE 2:**
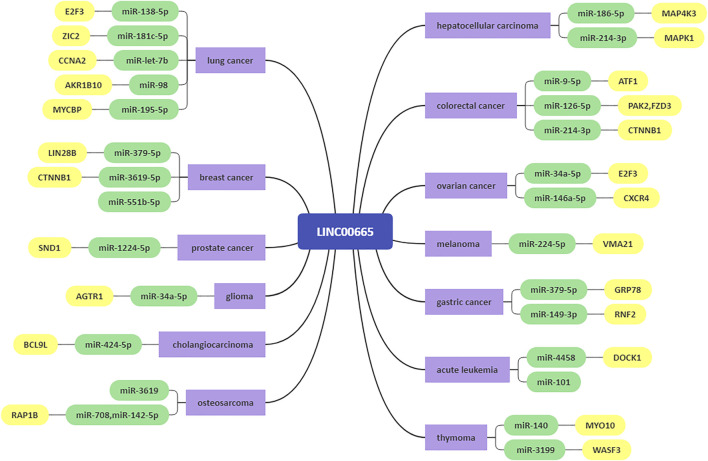
The ceRNA network of LINC00665.

**FIGURE 3:**
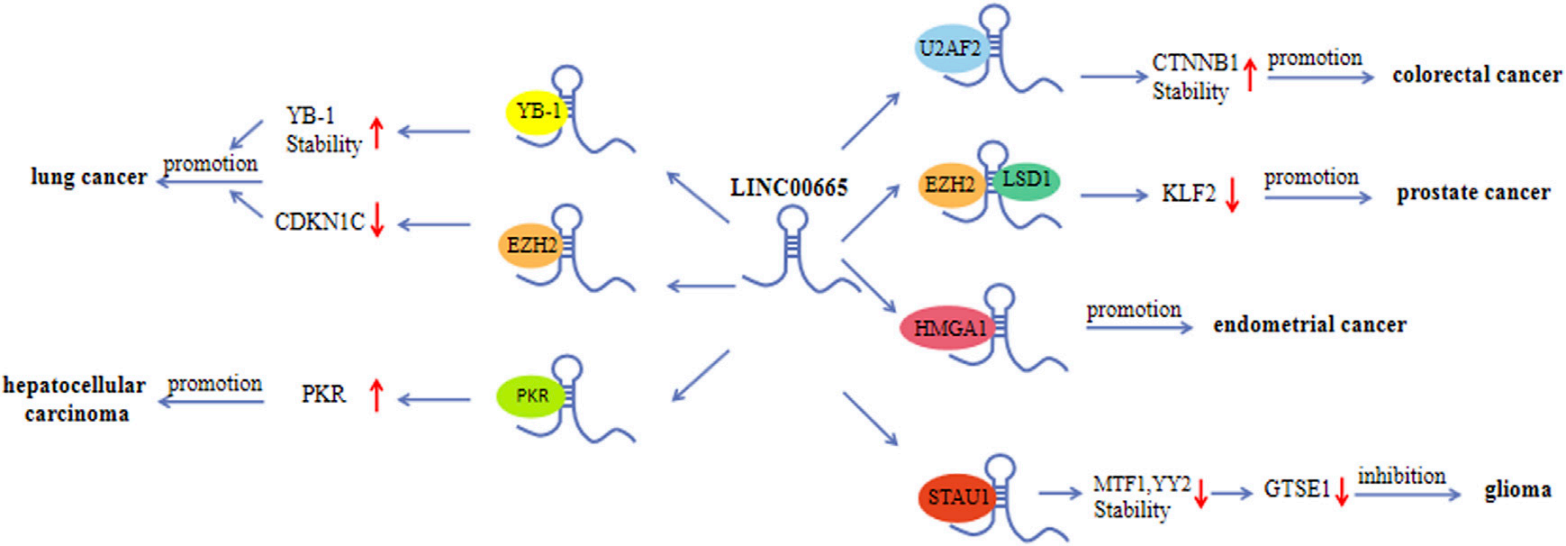
Interactions between LINC00665 and proteins.

**FIGURE 4:**
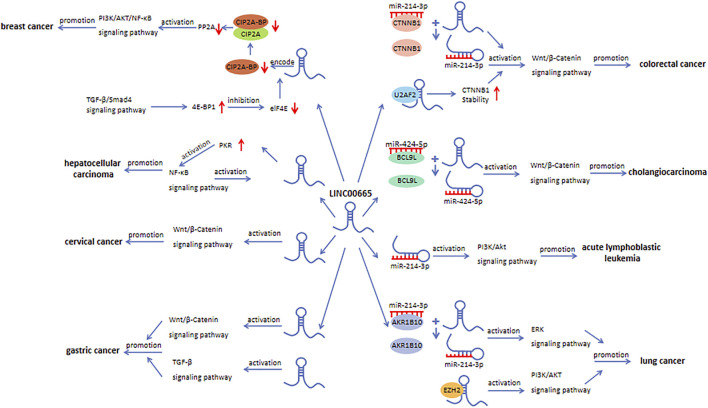
The role of LINC00665 in signaling pathways.

This article comprehensively summarizes the studies related to LINC00665 and systematically expounds on the expression level, biological function, molecular biological mechanisms, and clinical value of LINC00665 in human cancers. LINC00665 is abnormally expressed in multiple human cancers, and its dysregulation is significantly related to important clinical characteristics, such as tumor size, histological grade, TNM stage, and overall survival rate. In breast cancer and glioma, there are different conclusions about the expression level of LINC00665. This inconsistency may be caused by many factors, including the number of samples, the heterogeneity and specificity of the samples, and patients’ genetic and epigenetic differences. LINC00665-mediated regulation of cancer progression involves various mechanisms, including acting as a ceRNA (Figure 2), directly binding to and interacting with proteins (Figure 3), and being an upstream molecule that regulates multiple signaling pathways (Figure 4). As a novel lncRNA, LINC00665 has many unique features. Firstly, LINC00665 has a coding ability that most other lncRNAs do not have. Guo et al. showed that LINC00665 could encode a micropeptide called CIP2A-BP, thereby inhibiting the progression of triple-negative breast cancer. This discovery changes people’s traditional cognition and provides a new entry point for researching new cancer drugs. Secondly, LINC00665 plays a crucial role in various biological processes, including tumor cell proliferation, migration, invasion, apoptosis, autophagy, angiogenesis, and metabolism. Finally, abnormal expression of LINC00665 can not only affect the occurrence and development of cancers but also affect or predict the sensitivity of human cancers to chemoradiotherapy. These indicate that LINC00665 is a promising diagnostic, prognostic biomarker, and therapeutic target in terms of clinical application. Although researches on the biological functions of lncRNAs, including LINC00665, have made some progress, they are still in the preclinical stage, and there is still a long way to go before they are applied to the clinic.
